# Serum magnesium level and cardiac valve calcification in hemodialysis patients

**DOI:** 10.1186/s12872-023-03662-5

**Published:** 2023-12-13

**Authors:** Shihming Tsai, Yuehong Li, Xianglan Wu

**Affiliations:** Department of Nephrology, Beijing Tsinghua Changgung Hospital, School of Clinical Medicine, Tsinghua University, 168 Litang Road, Changping District, Beijing, 102218 China

**Keywords:** Hemodialysis, Hypomagnesemia, Magnesium, Cardiac valve calcification, Cardiovascular disease

## Abstract

**Introduction:**

Cardiac valve calcification is closely related to cardiovascular disease. The aim of this study was to investigate the magnesium level and cardiac valve calcification in hemodialysis patients.

**Methods:**

A cross-sectional study was conducted in 105 maintenance hemodialysis patients with complete follow-up data from June 2020 to May 2021 in Beijing Tsinghua Changgung Hospital, Tsinghua University. Baseline data, including sex, age, primary disease, liver and kidney function, electrolytes and parathyroid hormone, were recorded. According to their echocardiograms, patients were divided into a cardiac valve calcification group and a noncardiac valve calcification group, and the correlations between valve calcification and clinical data were analyzed.

**Results:**

Of 105 patients under hemodialysis, 60 (56.6%) were male, with an average age of 62.1 ± 13.5 years and a mean dialysis duration of 58.8 ± 45.4 months. The majority of primary renal diseases were diabetic nephropathy (55, 51.9%). Approximately 64.8% of the 105 maintenance hemodialysis patients had cardiac valve calcification, and 35.2% were in the noncardiac valve calcification group. The independent t test and the chi-square test analysis showed that the cardiac valve calcification group had older age, higher smoking rate, diabetes mellitus, lower extremity arterial occlusion, coronary heart disease, and coronary artery calcification ratio but lower parathyroid hormone, serum calcium, serum magnesium, albumin, prealbumin, and high-density lipoprotein cholesterol levels (*P* < 0.05). Logistic regression analysis showed that age, diabetes mellitus, coronary artery calcification, lower serum magnesium, lower serum calcium, and lower parathyroid hormone levels were associated with valve calcification.

**Conclusion:**

The presence of cardiac valve calcification was associated with age, calcium, phosphorus and lower magnesium level. These factors we should pay more attention in clinical practice.

## Introduction

 Cardiovascular disease is very common in end-stage renal disease (ESRD) patients, especially in maintenance hemodialysis (MHD) patients. Traditional risk factors include age, dialysis duration, smoking, diabetes mellitus (DM), and hypertension. Disorders of calcium and phosphorus metabolism, secondary hyperparathyroidism, and uremic toxins in MHD patients will increase the risk of atherosclerosis, vascular calcification, and all-cause mortality. The KDIGO guidelines recommend regular echocardiographic and cardiac valve calcification (CVC) risk factor assessments for patients with chronic kidney disease (CKD) stages 3–5. The risk of CVC increases with the patient’s dialysis duration, resulting in valvular heart disease. The effect of hypomagnesemia on vascular calcification in MHD patients has gradually received more attention. In this study, we aimed to analyze the correlation between serum magnesium levels and CVC in MHD patients and to provide a theoretical basis for its treatment and prevention.

## Materials and methods

This was a cross-sectional study of 105 MHD patients (age > 18 years; HD for ≥ 3 months; dialysis adequacy (Kt/V > 1.4)) who received hemodialysis at the Department of Blood Purification Center of Beijing Tsinghua Changgung Hospital from June 2020 to May 2021. The exclusion criteria were (1) serious infection; (2) heart failure; (3) severe cerebrovascular disease; (4) new-onset malignant tumor or life expectancy less than 6 months; and (5) inability to cooperate with the examination. All patients signed informed consent forms, and the study was approved by the Ethics Committee of Beijing Tsinghua Changgung Hospital, Tsinghua University.

Baseline data were collected, including age, sex, primary disease, dialysis duration, complications and medication. We also recorded the average data from blood samples drawn every month from June 2020 to May 2021. A Fresenius 4008s dialysis machine, FX80 dialyzer, arteriovenous fistula or tunneled central venous catheter was used as the dialysis pathway, blood flow > 200 ml/min, and dialysis fluid flow 500 ml/min. The dialysis liquid ingredients were as follows: sodium (Na) 138–140 mmol/L, potassium (K) 2.0–3.0 mmol/L, calcium (Ca) 1.25–1.5 mmol/L and magnesium (Mg) 0.5 mmol/L. The normal range of serum Mg concentration was 0.7–1.05 mmol/L, with < 0.7 mmol/L being hypomagnesemia and > 1.05 mmol/L being hypermagnesemia. Echocardiography was performed every 6 months using a Philips EPIQ7C machine by a single experienced investigator to evaluate the heart valve status. Valve calcification was defined as the presence of bright echoes exceeding 1 mm on one or more cusps of the valve [1]. An echo equal to or higher than the aortic echo was defined as a bright echo. We evaluated coronary calcification by chest CT. Patients were divided into a CVC group and a non-CVC group.

Statistical analysis was performed using SPSS software, version 25.0 (IBM Corp., Armonk, NY, USA). The measurement data are expressed as the mean ± SD, and categorical data are expressed as percentages or ratios. Data from the CVC group and non-CVC group were compared by the independent sample t test for measurement data and the chi-square test for categorical data. Logistic regression analysis was used to analyze the factors for CVC. P < 0.05 was considered statistically significant.

## Results

### General information

In total, 105 MHD patients were enrolled in the study: 60 (56.6%) were male, the average age was 62.1 ± 13.5 years, the mean dialysis duration was 58.8 ± 45.4 months, 28 (26.4%) smoked, 61 (57.5%) had DM, 19 (17.9%) had lower extremity arterial occlusion, and 56 (52.8%) had coronary heart disease. The majority of primary renal diseases were diabetic nephropathy (55, 51.9%), followed by chronic glomerulonephritis (15, 14.2%), hypertensive renal disease (14, 13.3%), polycystic kidney disease (6, 5.7%), IgA nephropathy (5, 4.7%) and membrane nephropathy (3, 2.8%). Of the 68 patients with CVC, 67 patients (63.8%) had aortic valve calcification (AVC), 26 (24.8%) had mitral valve calcification (MVC), 25 (23.8%) had both, and no patients had tricuspid valve calcification (TVC). The baseline laboratory indicators are shown in Table 1.

**Table 1:** The baseline data laboratory indicators

Items	Results
Male (%)	60 (56.6)
Age (years)	62.1 ± 13.5
Smoking (%)	28 (26.4)
Dialysis duration (months)	58.8 ± 45.4
DM (%)	61 (57.5)
LEAO (%)	19 (17.9)
CHD (%)	56 (52.8)
CAC (%)	58 (54.7)
CVC (%)	68 (64.8)
AVC (%)	67 (63.8)
MVC (%)	26 (24.8)
TVC (%)	0 (0)
AVC + MVC (%)	25 (23.8)
Combined tumor (%)	13 (12.4)
Kt/V	1.40 ± 0.27
GFR (ml/min/1.73 ^2^ )	4.71 ± 1.46
Serum UA (µmol/L)	454.1 ± 68.9
Serum Ca (mmol/L)	2.18 ± 0.15
Serum P (mmol/L)	1.89 ± 0.39
Serum K (mmol/L)	4.97 ± 0.51
Serum Na (mmol/L)	139.7 ± 2.7
Serum Mg (mmol/L)	1.11 ± 0.13
Hb (g/L)	117.4 ± 10.5
PTH (ng/L)	267.2 ± 154.7
ALP (U/L)	80.8 ± 32.8
TP (g/L)	65.5 ± 4.7
Alb (g/L)	40.1 ± 2.8
Pre-Alb (mg/L)	324.8 ± 60.7
TC (mmol/L)	3.70 ± 1.04
TG (mmol/L)	2.05 ± 1.15
LDL-C (mmol/L)	1.96 ± 0.85
HDL-C (mmol/L)	0.93 ± 0.27
Drug	
Calcium carbonate (%)	33 (31.1)
Lanthanum carbonate (%)	14 (13.2)
Sevelamer (%)	43 (40.6)
Cinacalcet (%)	20 (18.9)
Calcitriol or Paricalcitol (%)	50 (47.2)

*DM* Diabetes mellitus, *LEAO* Lower extremity arterial occlusion, *CHD* Coronary heart disease, *CAC *Coronary artery calcification, *GFR* Glomerular filtration rate, *UA *Uric acid, *Ca *Calcium, *P *Phosphorus, *K *Potassium, *Na *Sodium, *Mg*  Magnesium, *Hb *Hemoglobin, *PTH *Parathyroid hormone, *ALP *Alkaline phosphatase, *TP *Total protein, *Alb * Albumin, *TC *Total cholesterol, *TG *Triglycerides, *LDL-C *Low-density lipoprotein cholesterol, *HDL-C *High-density lipoprotein cholesterol

### Clinical data compared between the CVC group and the non-CVC group

Of the 105 patients, 68 (64.8%) had CVC, and 37 (35.2%) did not. The independent t test and chi-square test analysis showed that the CVC group was older and had higher rates of smoking, DM, lower extremity arterial occlusion, CHD, and CAC but lower PTH, serum Ca, Mg, Alb, pre-Alb, and HDL-C levels than in non-CVC group (*P* < 0.05). See Table 2.

**Table 2:** Comparison between the CVC group and non-CVC group

Items	CVC group (*n* = 68)	Non-CVC group (*n* = 37)	*P *value
Age (years)	66.5 ± 10.1	53.9 ± 15.4	0.000^*^
Male (%)	42 (61.7)	18 (48.6)	0.194
Smoking (%)	23 (33.8)	5 (13.5)	0.025^*^
Dialysis duration (months)	58.3 ± 44.9	59.7 ± 46.7	0.881
DM (%)	50 (73.5)	11 (29.7)	0.000^*^
LEAO (%)	18 (26.4)	1 (2.7)	0.003^*^
CHD (%)	43 (63.2)	13 (35.1)	0.006^*^
CAC (%)	45 (66.1)	13 (35.1)	0.002^*^
Combined tumor (%)	13 (19.1)	0 (0)	0.004
Kt/v	1.39 ± 0.28	1.41 ± 0.26	0.804
GFR (ml/min/1.73^2^)	4.93 ± 1.58	4.31 ± 1.12	0.040^*^
Serum UA(µmol/L)	449.5 ± 63.2	462.6 ± 78.5	0.357
PTH (ng/L)	241.8 ± 113.8	313.7 ± 203.9	0.022^*^
ALP (U/L)	85.2 ± 35.8	72.7 ± 24.8	0.064
Serum Ca (mmol/L)	2.15 ± 0.13	2.23 ± 0.14	0.007^*^
Serum P (mmol/L)	1.84 ± 0.40	1.98 ± 0.36	0.084
Serum K (mmol/L)	4.97 ± 0.53	4.97 ± 0.49	0.936
Serum Mg (mmol/L)	1.09 ± 0.14	1.15 ± 0.12	0.025^*^
Hb (g/L)	117.2 ± 10.5	117.9 ± 10.5	0.750
TP (g/L)	65.7 ± 4.9	65.1 ± 4.3	0.532
Alb (g/L)	39.7 ± 2.8	40.8 ± 2.6	0.049^*^
Pre-ALb (mg/L)	310.8 ± 58.1	350.6 ± 57.5	0.001^*^
TC (mmol/L)	3.69 ± 1.04	3.74 ± 1.05	0.813
TG	2.12 ± 1.22	1.91 ± 1.02	0.372
LDL-C (mmol/L)	1.96 ± 0.87	1.95 ± 0.82	0.947
HDL-C (mmol/L)	0.88 ± 0.26	1.03 ± 0.26	0.006^*^
Drug			
Calcium carbonate (%)	21 (30.9)	12 (32.4)	0.870
Lanthanum carbonate (%)	7 (10.3)	7 (18.9)	0.214
Sevelamer (%)	27 (39.7)	16 (43.2)	0.725
Cinacalcet (%)	9 (13.2)	11 (29.7)	0.004^*^
Calcitriol or Paricalcitol (%)	29 (42.6)	21 (56.7)	0.167

*P<0.05

### Logistic regression analysis of CVC risk factors

To identify factors correlated with CVC, we performed further logistic regression analysis, and the results showed that age, DM, CAC, and lower serum Mg, Ca, and PTH levels were associated with CVC. All *P* < 0.05, see Table 3.

**Table 3:** Logistic regression analysis of CVC

Items	*OR*	95% *CI*	*P* value
Serum Mg	0.010	0.000 –0.754	0.037
Serum Ca	0.008	0.000 –0.621	0.030
PTH	0.996	0.993 –1.000	0.032
Age	1.107	1.045–1.172	0.001
DM	3.902	1.256–12.119	0.019
CAC	3.397	1.093–10.556	0.035

## Discussion

Cardiovascular conditions such as CHD, arrhythmia, and sudden cardiac death are common in patients with ESRD. The incidence of cardiovascular disease in MHD patients is 8 times that in the general population [2]. The progression of vascular calcification in ESRD patients is 10 times faster than that in the general population [3]. Vascular calcification and CVC increase the risk of cardiovascular disease, and common risk factors include age, DM, hypertension, smoking, and Ca-P metabolism disorders. The CVC in patients with CKD is mainly AVC and MVC, with a prevalence of approximately 25-59%, and CVC arises approximately 10–20 years earlier in CKD patients. The annual incidence of AVC in MHD patients is close to 3.3%, and it increases with the decline in GFR [3, 4]. Most (63.8%) of our patients had AVC, followed by MVC (24.8%). Vascular calcification in MHD patients is related to a variety of factors, such as dialysis duration, inflammatory factors, poor nutritional status, hyperlipidemia, Ca-P metabolism disorders, and secondary hyperparathyroidism [5]. Our study found that the CVC group was older, more often smoked, more often had DM and more often had lower extremity arterial occlusion history but had lower HDL-C. Calcium deposits in the valve and abnormal metabolism of blood glucose, lipids and lipoproteins will thicken and harden the arterial wall, reduce its elasticity, narrow the vascular lumen, and increase the risk of vascular calcification. Therefore, these CVC groups are more often complicated with lower extremity arterial occlusive disease.

Vascular calcification in ESRD patients mainly occurs in the vascular media, suggesting the existence of passive mineral deposition and active cell activation processes [5]. CVC is associated with the risk and severity of CHD in predialysis CKD patients [6]. It has been reported that CVC in CKD patients is associated with myocardial ischemia, suggesting that AVC and MVC are predictors of CHD [6]. A previous study showed that CVC in dialysis patients was associated with all-cause mortality [7]. The 1-year all-cause mortality in MHD patients with both aortic and mitral valve calcification is 57%, in those with either valve calcification is 40%, and in those with neither valve calcification is 15% [8]. Japanese and Chinese cohort studies also found that valve calcification in MHD patients could predict cardiovascular mortality and all-cause mortality [9, 10]. Patients with hypoalbuminemia have poor immunity and are prone to inflammation. Studies have found that hypoalbuminemia is an independent risk factor of CVC [11]. Our study showed that the CVC group had a high percentage of CHD and CAC. For MHD patients without symptoms of CHD, CVC is an important indicator for predicting and assessing the risk of CHD; therefore, it should be assessed as early as possible for long-term prognosis.

Mg is an important cation in physiological processes in the human body. In recent years, the effect of serum Mg levels on vascular calcification in dialysis patients has received attention. Three main Mg concentrations are used in the dialysate: 0.25 mmol/L, 0.5 mmol/L and 0.75 mmol/L. Serum Mg levels were mainly affected by the difference between serum and dialysate Mg concentrations and the Gibbs–Downan effect. The use of low-Mg dialysate and lower serum albumin levels can easily cause hypomagnesemia. In addition to traditional risk factors, vascular calcification inhibitory factors (such as matrix Gla protein and bone morphogenetic protein-7) and promotion factors (such as higher P and Ca levels, inflammatory status, oxidative stress, and uremic microenvironment) are also reasons for vascular calcification [12]. Previous studies have found that Mg can inhibit the Wnt/B-catenin signaling pathway and inhibit vascular calcification; therefore, maintaining a slightly higher level of serum Mg can delay the progression of vascular calcification [13].

One study found that Mg level could predict mitral annular calcification in MHD patients, and hypermagnesemia might prevent mitral annular calcification [14]. Hypomagnesemia in MHD patients increases oxidative stress and inflammation, the mechanism of which is closely related to atherosclerosis and vascular calcification, resulting in a high risk of cardiovascular disease and high all-cause mortality. In this study, dialysate with 0.5 mmol/L Mg was used. The levels of serum Mg and albumin in the CVC group were lower, the serum Ca and PTH levels were slightly lower than those in the non-CVC group, and there was no significant difference in serum P. Logistic regression analysis showed that lower Mg level was a related factor for CVC. Possible mechanisms are that lower Mg level causes an imbalance between calcification inhibitors and promoters and increases oxidative stress and inflammatory responses, resulting in valve calcification.

The formation and progression of CVC are influenced by serum Ca, P and PTH. Maintaining the balance of the three is very important to prevent the risk of CVC in MHD patients. Calcium-containing phosphorus binders increase the risk of CVC. A randomized controlled trial in 186 MHD patients compared the progression of CVC when patients received calcium-containing phosphorus-binding agents or sevelamer. The results showed that sevelamer reduced the progression of CVC [15]. A meta-analysis showed that non-Ca-containing phosphorus binders can reduce mortality in patients with CKD or ESRD [16]. The ADVANCE study in 360 MHD patients with secondary hyperparathyroidism, divided into a calcimimetic (cinacalcet) combined with low-dose vitamin D group and a vitamin D alone group, found that low-dose vitamin D combined with cinacalcet delayed the progression of CVC [17]. In our study, more patients used cinacalcet (29.7%) than in the CVC group (13.2%), suggesting that cinacalcet reduces PTH levels and the release of Ca and P from bone, resulting in a reduced dose of vitamin D.

Limitations of our study were the single-center cross-sectional design and small sample size. We also did not collect data on parathyroidectomy, drug dosage, statin usage or not and the type of hemodialysis. Therefore, we look forward to multicenter, large-sample, prospective cohort studies to further explore this issue.

## Conclusion

The presence of cardiac valve calcification was associated with age and calcium, phosphorus, and lower magnesium level. Magnesium plays an important role in cardiac valve calcification in hemodialysis patients. Paying greater attention to serum Mg levels and maintaining Mg, Ca, P and PTH at suitable levels can be beneficial for patients.

## Data Availability

All data generated or analyzed during this study are included in this article. Further enquiries can be directed to the corresponding author.
